# Experimental Analysis of the Enzymatic Degradation of Polycaprolactone: Microcrystalline Cellulose Composites and Numerical Method for the Prediction of the Degraded Geometry

**DOI:** 10.3390/ma14092460

**Published:** 2021-05-10

**Authors:** Jacob Abdelfatah, Rubén Paz, María Elena Alemán-Domínguez, Mario Monzón, Ricardo Donate, Gabriel Winter

**Affiliations:** 1 Mechanical Engineering Department, Universidad de Las Palmas de Gran Canaria, 35017 Las Palmas, Spain; jacob.abdelfatah@ulpgc.es (J.A.); mariaelena.aleman@ulpgc.es (M.E.A.-D.); mario.monzon@ulpgc.es (M.M.); ricardo.donate@ulpgc.es (R.D.); 2 Institute of Intelligent Systems and Numerical Applications in Engineering (SIANI), Universidad de Las Palmas de Gran Canaria, 35017 Las Palmas, Spain; gabriel.winter@ulpgc.es

**Keywords:** enzymatic degradation, polycaprolactone, numerical method, prediction of degraded geometry, Monte Carlo method, microcrystalline cellulose

## Abstract

The degradation rate of polycaprolactone (PCL) is a key issue when using this material in Tissue Engineering or eco-friendly packaging sectors. Although different PCL-based composite materials have been suggested in the literature and extensively tested in terms of processability by material extrusion additive manufacturing, little attention has been paid to the influence of the fillers on the mechanical properties of the material during degradation. This work analyses the possibility of tuning the degradation rate of PCL-based filaments by the introduction of microcrystalline cellulose into the polymer matrix. The enzymatic degradation of the composite and pure PCL materials were compared in terms of mass loss, mechanical properties, morphology and infrared spectra. The results showed an increased degradation rate of the composite material due to the presence of the filler (enhanced interaction with the enzymes). Additionally, a new numerical method for the prediction of the degraded geometry was developed. The method, based on the Monte Carlo Method in an iterative process, adjusts the degradation probability according to the exposure of each discretized element to the degradation media. This probability is also amplified depending on the corresponding experimental mass loss, thus allowing a good fit to the experimental data in relatively few iterations.

## 1. Introduction

Polycaprolactone (PCL) is a polyester that has been widely used for medical applications [1,2] and as a substitute of oil-derived thermoplastics for packaging [3,4]. In both fields, biodegradability is one of the key features of the material, so it would be desirable to be able to modulate the biodegradation rate in order to fulfil versatile requirements. The in vivo degradation of PCL may require 2–3 years [5], which is a remarkably slower rate than the values reported for similar materials commonly used in Tissue Engineering applications, such as polylactic acid (PLA) or polyglycolic acid (PGA), because of its higher hydrophobicity [6,7]. Indeed, this period of time may be excessive for some specific applications in the Tissue Engineering field, where the degradation rate should match the growth rate of the surrounding tissue [5,8].

In the process of polymer degradation, chain cleavage is referred to as degradation, and the loss of material mass as erosion [9]. The primary mode of degradation is chain cleavage through hydrolysis, either through abiotic (non-enzymatic) hydrolysis or enzyme-promoted hydrolysis. The hydrolytic degradation of PCL takes place through the cleavage of the ester bonds in the structure through a complex process that depends on the molecular weight of the polymer, its crystallinity and the shape of the structure to be degraded, among other factors [7]. Enzymatic degradation has been reported to increase the degradation rate of the material by a similar mechanism to the one reported for the hydrolytic degradation [10]. In particular, lipases have demonstrated to catalyze the hydrolytic degradation of PCL [11,12,13], so they can be used in order to analyze the changes in the degradation of PCL-based materials with reduced observation periods. In addition to this, lipase-mediated degradation is one of the key elements to explain the compostability of this material [14], so the analysis of this type of process has a remarkable importance for the development of materials with tailored degradation rate values. As enzymes are too bulky to penetrate into the polymer matrix, enzymatic hydrolysis is mainly a surface erosion process [15], and the resulting polymer erosion depends on many factors, including polymer degradation, swelling, crystallinity, hydrophobicity, polymer chain length and water diffusivity [16].

Different strategies have been explored in order to modify the degradation rate of PCL materials without modifying their advantages as functional biodegradable thermoplastics, such as the development of blends, copolymers [6,7] and composites. The formulation of composites by introducing a filler in a PCL matrix can modify the degradation properties of the base material, as the presence of the filler may vary the crystallinity of the matrix [17] and this parameter has a great influence on the degradation mechanism of the material [17,18]. In addition to this, the presence of fillers can change the surface properties of the material, including its roughness [19] and wettability (i.e., Yin et al. demonstrated that bioglass increases water uptake values of PCL-based scaffolds [20]). These changes can modify the enzyme adsorption rate, which is the rate-determining step of the enzymatic degradation mechanism of polyesters.

Although fillers have been widely analyzed in terms of bioactivity improvement of PCL-based matrices, normally this type of studies do not focus on the possibility of using this strategy as a way of tailoring the degradation kinetics of the base materials [3]. The currently available publications on the issue usually cover the degradation of PCL composites by composting in soil [7], or their in vitro degradation when shaped as specific structures to be used in Tissue Engineering applications [8,21]. However, there is limited data related to controlled enzymatic degradation analysis for PCL-based composites or its modelling with standardized samples. One of the scarce references on the topic is the work described by Nerantzaki et al. [22], who demonstrated that the presence of different nanofillers delay the enzymatic degradation of the PCL matrix. However, the trend is not universal and it depends on the type of filler and its dispersion, among other factors.

Microcrystalline cellulose (MCC) is a renewable material which can be used to obtain PCL-composite materials feasible to be applied as feedstock in material extrusion Additive Manufacturing processes. In fact, in previous studies, the authors demonstrated that polycaprolactone:microcrystalline cellulose (PCL:MCC) blends containing 2% wt:wt of MCC can be processed to obtain scaffolds with promising features to be used in Bone Tissue Engineering [23], but they can also be interesting as an eco-friendly alternative to current filaments in the manufacturing of 3D printed structures for other fields. In this work, the filaments used as feeding material during the extrusion-based 3D printing have been analyzed instead of the final structures, as the information from this assessment could be used in order to simulate the properties of any structure by FEA (Finite Element Analysis) [24]. Besides, these data provide a more accurate assessment of the material, as the evaluation of the degradation pathways of 3D printed porous structures will be affected by the design of the inner porosity of the analyzed samples [25], and the results would have limited impact on the prediction of the behavior of different types of parts. In addition to this, the extrusion process to obtain the filament may affect the crystallinity of the material [26] and, as a consequence, its degradation profile. Limited analysis of the degradation of composite materials to be used in extrusion-based 3D printing processes have been carried out and none has been found regarding the evolution of the mechanical properties during the enzymatic degradation.

The results of this work could be exploitable both in the development of eco-friendly commercial 3D-printed structures and in the Tissue Engineering field, as the extracellular in vivo degradation and the degradation in soil are similar in their initial steps. Only after reaching values below 10 kDa [3] does the intracellular degradation take place and the mechanisms start to diverge. As a consequence, the possibility of tailoring the first steps of PCL degradation has a great impact both for environmental and Tissue Engineering applications.

On the other hand, different models have been proposed to predict the degradation process of polymers. Among them, there are two main groups apart from the empirical methods: the phenomenological and probabilistic methods [27].

The phenomenological models are deterministic methods which model the process of degradation using governing equations such as reaction diffusion or Flick Law. In this regard, Wang and his coworkers considered a model in which the degradation process occurs due to a hydrolytic process in which the ester back bonds are cleaved by the diffusion of the water molecules inside of the structure [28]. Later, Sevim and Pam introduced the autocatalytic effect and coupled the reaction diffusion equation to a Monte Carlo based erosion model [29]. Despite both models showing good agreement, they only considered the degradation due to the pure hydrolytic process.

Regarding the probabilistic methods, Gopferich et al. developed a method that considered the crystallinity and amorphous areas, previously generated and implemented into a Poisson probability distribution function, and applied Monte Carlo to simulate the degradation process [16]. The main disadvantage of this methodology is that it can only be applied in simple geometries [30]. However, Erkizia et al. [31] proposed a new method, also based on Monte Carlo, which could deal with complex geometries, such as scaffolds. They discretized the initial geometry in voxels and the degree of exposure of each voxel to the liquid was used as the main parameter to drive the degradation (the higher the exposure to the liquid, the higher the degradation probability), obtaining a good agreement with the experimental data.

In this work, a new hybrid experimental-probabilistic model is proposed to take into account the experimental mass loss (by the effect of enzymatic hydrolysis of the material) as reference values to adjust the probability function (also based on Monte Carlo method). This allows a good agreement with the experimental results, which can be useful to predict the degraded geometry according to the experimental data. Moreover, the results could be easily extrapolated to any other geometry with the same material and conditions to predict the degraded shape at the different degradation times.

## 2. Materials and Methods

### 2.1. Materials and Experimental Tests

Polycaprolactone (PCL) Capa^®^ 6800 with a mean molecular weight of 80,000 Da, melting point of 58–60 °C and melt flow index of 3 g/10 min (with 2.16 kg and 1″ PVC die at 160 °C) was kindly supplied by Perstorp, Warrington, UK. Microcrystalline cellulose (MCC) with 51 um particle size and 0.6 g/mL bulk density was purchased (Sigma Aldrich, Munich, Germany). PCL was ground at 8000 rpm in an Ultra Centrifugal Mill ZM Retsch device (RETSCH, Haan, Germany). This material was mixed with microcrystalline cellulose to obtain a mixture containing 2% wt:wt of MCC (PCL:MCC 98:2). Sheets of composite material were obtained by compression molding in a Collin P 200 P/M press (Collin Lab & Pilot Solutions, Maitenbeth, Germany) at 85 °C and a pressure of 10 bar. The compression molding sheets were cut to obtain pellets. These pellets were extruded in an extrusion device with a 8 mm cylinder, a L/D ratio of 10 and a nozzle tip of 1.5 mm (cylindrical inner shape), as described in [32]. The extrusion temperature was 120 °C (extrusion device with one heating zone) and the rotational speed of the screw, 7 rpm. In the case of pure PCL, the commercial pellets were directly extruded in this extrusion device. In both cases, the filament obtained had 1.9 mm diameter.

Fragments of approximately 50 mm length of these filaments (PCL and PCL:MCC 98:2) were subjected to degradation with a 7 mg/mL suspension of lipases from Pseudomonas cepacia (Sigma Aldrich) in PBS 2× (Sigma Aldrich). Different degradation periods were analyzed: 4, 24 and 48 h. After these time periods, the samples were rinsed with distilled water and ethanol, and allowed to dry. Afterwards, they were weighed (±0.0001 g) and then tested (tensile test) in a LY-1065 Universal testing machine (Dongguan Liyi Environmental Technology Co., Ltd., Dongguan, China) with a gauge length of approximately 30 mm and crosshead speed of 2 mm/min. The weighing and tensile tests were carried out at room conditions (approximately 20 °C and 60% relative humidity). 4 replicas per group were used (mass of the initial samples: 0.1584 ± 0.0189 g for PCL and 0.1613 ± 0.0163 g for PCL:MCC 98:2).

In addition, the pH value of the degradation media was measured with a Hach pHmeter (±0.01 pH units, Hach, Loveland, CO, USA). The morphology of the samples was also observed by scanning electron microscopy (SEM) in a Hitachi TM3030 microscope (Hitachi, Tokyo, Japan). Before observation, the samples were sputtered with a mini sputter coater (Quorum technologies SC7620 model, Laughton, UK) with an Au/Pd target. The metallization process took place at 18 mA of intensity for 120 s.

Finally, the FTIR spectra of the samples at 0, 24 and 48 h was obtained in a Perkin Elmer IR Spectrum Two device (PerkinElmer, Inc., Waltham, MA, USA) in the ATR (Attenuated Total Reflection) mode between 450 and 4000 cm^−1^ resolution. The area of the CH_2_ peak at 2945 cm^−1^ and the C–O–C peak at 1245 cm^−1^ were measured with the Spectum 10^TM^ software (Spectrum 10 STD, PerkinElmer, Inc., Waltham, MA, USA) in order to evaluate the ratio of the values and, hence, the relative amount of amorphous and crystalline regions [23].

### 2.2. Numerical Method for the Prediction of the Degraded Geometry

**Figure 1:**
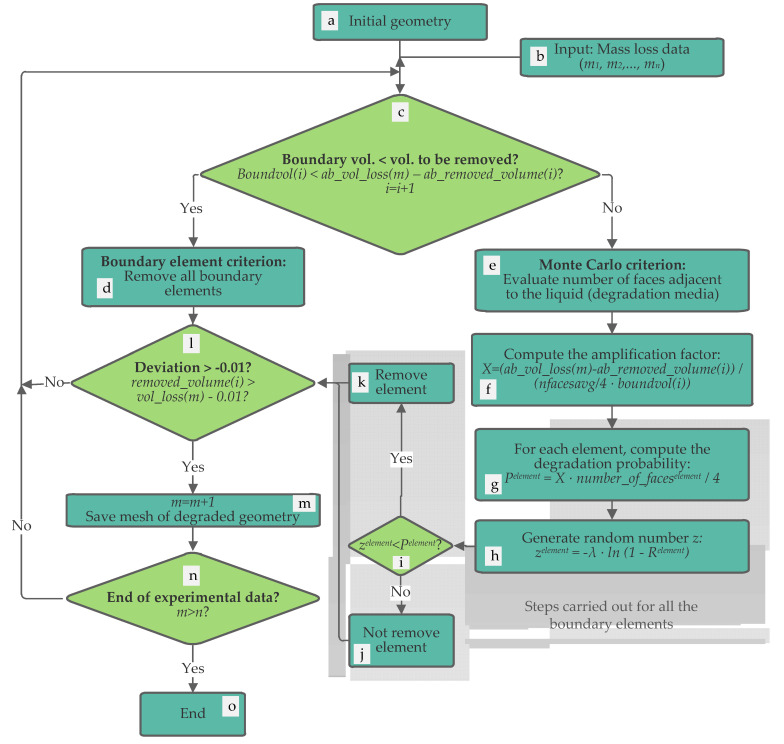
Flow chart of the proposed method to simulate the enzymatic degradation using the experimental data of mass loss as reference values to adjust the degradation and predict the degraded geometry.

The proposed methodology for the prediction of the degraded geometry is summarized in Figure 1. This methodology was implemented in FreeFEM++ software (version 4.5, Frédéric Hecht (laboratory Jacques-Louis Lions), Paris, France) [33]. The first step consists in generating the initial geometry to be analyzed (degraded). This geometry is meshed (in this case, using the Gmsh software (version 4.6, Christophe Geuzaine (University of Liège) and Jean Francois Remacle (Catholic University of Louvain), Liège/Louvain, Belgium [34]) and loaded into the program (Figure 1a). Additionally, the experimental mass loss data are added as input parameters (Figure 1b) in the algorithm (percentage values of mass loss, which correspond to different degradation times). The method assumes a constant density, which means that the percentage of mass loss (experimental data) and the percentage of volume loss are the same (hereinafter, both mass loss and volume loss, in percentage, will be the same).

Once the previous steps are completed, the algorithm evaluates if the volume of the boundary elements is enough to reach the reference volume loss (absolute value, in mm^3^, obtained from the first experimental data, in percentage) (Figure 1c). To do this, the method compares the volume of the boundary elements (boundvol(i), mm^3^) with the difference between the volume already removed (ab_removed_volume(i), in mm^3^) and the next volume reference (ab_vol_loss(m)), which, at the beginning, corresponds to the first experimental mass loss data but in absolute value (mm^3^):

Boundvol(i) > ab_vol_loss(m) − ab_removed_volume(i),(1)

‘i’ being the iteration number and ‘m’ a counter for the experimental data.

If this previous condition is not fulfilled, the boundary elements are removed (Figure 1d, boundary element criterion). However, if the previous condition is fulfilled, the Monte Carlo criterion is applied (Figure 1e). Note that in each iteration, only the boundary elements will be evaluated, which is in line with the enzymatic process (mainly a surface erosion process). In the case of application of the Monte Carlo criterion, a similar approach to that developed by Erkizia et al. [31] is applied. In that work, the normalized influence of the faces and edges of liquid voxels in contact with the liquid was considered to determine the degradation probability. In this new methodology, however, a new parameter is added (amplification factor, X) to adjust the degradation probability to the experimental mass loss, thus accelerating the convergence of the method to the experimental results. Therefore, the first step is to evaluate the number of faces adjacent to the degradation media. It consists of evaluating, for each contour element, the number of faces that are in direct contact with the liquid. The elements with at least one face without adjacent elements will be considered as boundary elements (e). In this step, all the boundary elements are labelled and the average number of faces in contact with the liquid (nfacesavg) is also calculated.

The next step (f) consists of computing the amplification factor (X). This factor takes into account the reference value of volume loss (ab_vol_loss(m), in mm^3^), the already removed volume (ab_removed_volume(i), in mm^3^), the average number of faces in contact with the liquid (nfacesavg) and the volume of the boundary elements (bundvol(i), also in mm^3^):

X = (ab_vol_loss(m) − ab_removed_volume(i))/(nfacesavg/4 · boundvol(i)),(2)

The amplification factor represents the proportion of volume of the boundary elements that should be removed to achieve the reference experimental value of mass loss, but divided by the average number of faces (boundary elements) in contact with the media. This means that the higher this value is, the higher the volume that must be removed from the boundary elements to reach the experimental data.

After the amplification factor is determined, the algorithm evaluates the degradation probability (P^element^) of all the boundary elements as follows (Figure 1g):

P^element^ = X · number_of_faces^element^/4, (3)

where ‘number_of_faces^element^’ is the number of faces of the element in contact with the liquid or degradation media. In other words, the probability of degradation of each element depends on the ratio of number of faces in contact with the media with respect to the total number of faces (4), multiplied by the amplification factor (X).

Within the loop that computes the degradation probability, a random number (z^element^) is also generated (Figure 1h) [35]:

Z^element^ = −λ·ln (1 − R^element^), (4)

‘λ’ being a parameter that can be modified to slow down or speed up the degradation rate (in this case λ = 0.00812), and ‘R^element^’ a random number obtained from a uniform distribution.

Therefore, for each boundary element, the degradation probability is determined and a random number is generated. Then, both values are compared (Figure 1i), and if the random number (z^element^) is lower than the degradation probability (P^element^), the corresponding element is removed (Figure 1k). Otherwise, the element is not removed (Figure 1j).

The following step (l in Figure 1) is common for both criteria (boundary element and Monte Carlo). In this stage, the method evaluates the deviation between the simulated removed volume (removed_volume(i)) and the reference experimental (removed_volume(i)), both in percentage values, allowing a minimum difference of −0.01%. For higher values, the condition is fulfilled:

removed_volume(i) > vol_loss(m) − 0.01.(5)

If the deviation is higher than −0.01% (Figure 1m), then algorithm saves the resulting mesh (which will correspond to the degraded geometry at the degradation time corresponding to the m counter). For example, when the m counter is increased the first time, the resulting degraded mesh would correspond to the degraded geometry at the first degradation time, which is 4 h in this case. Additionally, when this criterion is fulfilled, the ‘m’ counter is increased so that the next value of mass loss from the experimental results will be selected as reference value for the following iterations. However, if the removed volume is still too low compared with the reference value, then the deviation will be lower than −0.01% and this criterion will not be fulfilled. In this case, the algorithm repeats the process (starting from c in Figure 1).

This methodology is repeated in a loop while the ‘m’ counter is not higher than the available experimental data (n) (n in Figure 1). Otherwise, the simulation is finished (o in Figure 1).

**Figure 2:**
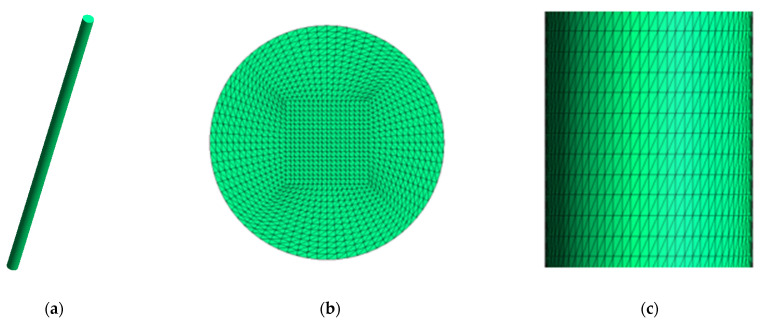
Design of the filament (1.9 mm diameter and 50.3 mm length) and partial views of the meshed geometry (with 4,258,800 elements and 730,431 nodes): (**a**) Initial geometry of the filament; (**b**) Cross section of the discretized geometry; (**c**) Longitudinal cut of the discretized geometry.

This methodology was applied in two different cases (PCL and PCL:MCC 98:2 filaments). In both cases, the initial design of the geometrical model was the same, corresponding to the measurements of the real filaments used in the experimental tests (1.9 mm diameter and 50.3 mm length). A cylindrical geometry was designed with these values and the resulting geometry was imported in Gmsh software [34] to generate the mesh. The geometry was discretized in tetrahedral elements, obtaining the structured mesh grid depicted in Figure 2, with a total of 4,258,800 tetrahedrons and 730,431 nodes.

This mesh was loaded in the FreeFEM++ [33] developed algorithm. Additionally, the experimental mass loss data (percentage values) were added as input values according to Table 1. Both the degradation of the PCL and PCL:MCC filaments were simulated in a cluster with 28 compute nodes and an additional one for access. The compute nodes are grouped into seven Bullx R424E2 computers, each one with four compute nodes. Each compute node consists of:

- A total of 2 Intel Xeon E5645 Westmere-EP processors, with 6 cores for each one.
- A total of 48 GB of RAM.
- A 500 GB hard drive
- Infiniband interface at 40 Gbs.

## 3. Results 

### 3.1. Mass Loss and Mechanical Properties

As shown in Table 1, the presence of MCC accelerates the mass loss of the samples. By the end of the longest experiment (48 h), the reported value was 5.88 ± 0.28% for pure PCL, while it was 16.15 ± 5.30% for the composite samples (2.7 times higher). The increase in the mass loss percentage is higher than the concentration of microcrystalline cellulose (2% wt:wt), so it is evident that the degradation rate of the matrix is affected by the presence of the filler.

The decrease in pH evolves according to the results of mass loss: the highest reduction was found for the composite samples (showing a final value of 4.35 compared to the value of 5.59 found for pure PCL samples), as carboxylic acids are released during the degradation and these compounds are able to decrease the pH of the environment [23]. An increment in the local concentration of these acids can promote an autocatalytic effect [23] and, hence, accelerate the hydrolytic degradation of the structure. On the other hand, it should be noted that the specific activity of lipases from Pseudomonas cepacia is, at least, 30 U/mg (being 1 U the amount of enzyme which liberates 1 μmol oleic acid per minute at pH 8.0 and 40 °C). At different pH conditions, this specific activity may be lower (thus influencing on the degradation rate), but according to the literature [36], lipase can perform properly at different ranges of pH.

The results from the mechanical characterization are shown in Table 1. At the beginning of the degradation process, composite samples provided lower values of the elastic modulus (295 ± 17 MPa) than pure PCL samples (306 ± 46 MPa). During the experiment, it was possible to observe a higher decrease in the value of this parameter for PCL:MCC 98:2 (27%) than for pure PCL (12%). This higher reduction in the mechanical properties of the composite material can be explained to some extent by the higher values of mass loss reported in the third column in Table 1. In the case of PCL, the mass loss and elastic modulus did not significantly change between 24 and 48 h. In fact, both parameters slightly increased, probably due to enzyme saturation, which limited the enzymatic degradation.

**Figure 3:**
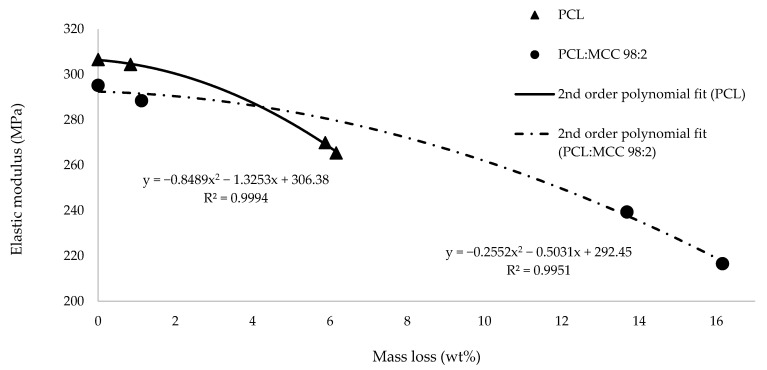
Evolution of the modulus of PCL and composite material with degradation time.

For this reason, it is relevant to analyze the evolution of the elastic modulus with the mass loss (Figure 3). When these two variables are compared, it is possible to observe that the loss in elastic modulus depends more abruptly on mass loss for pure PCL than for the composite material.

### 3.2. SEM Observation

**Figure 4:**
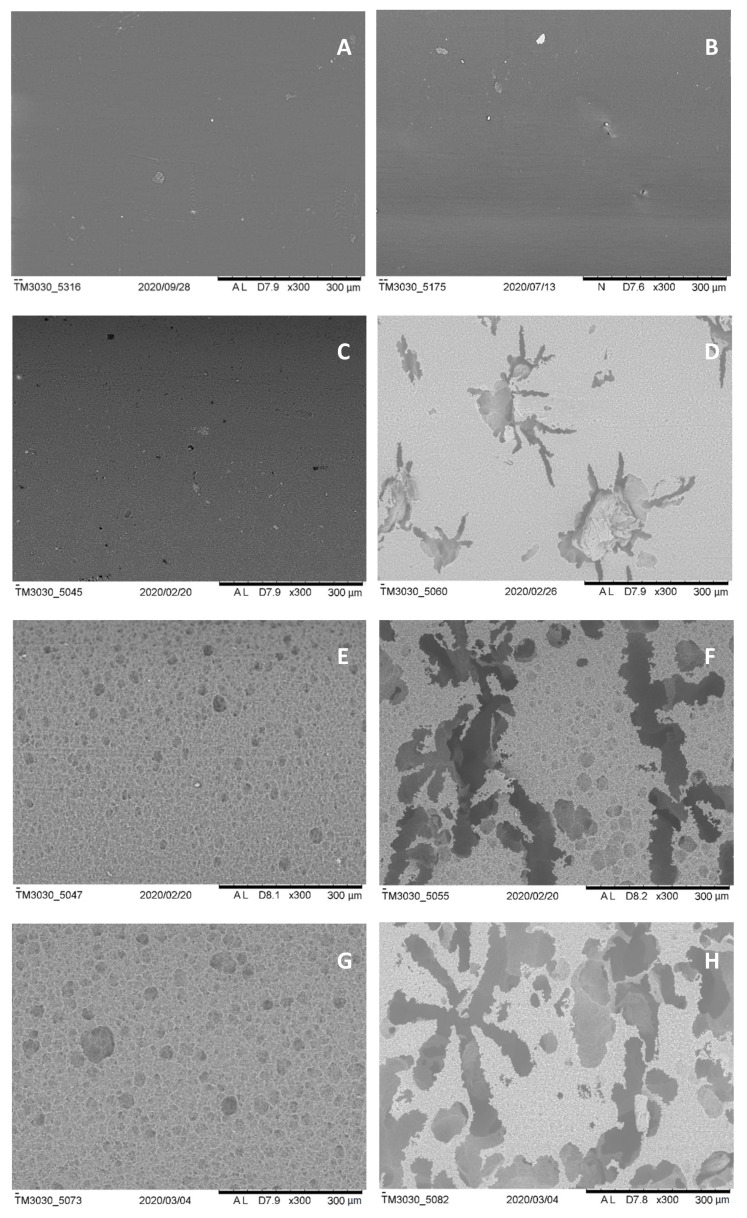
(**A**) PCL and (**B**) PCL:MCC 98:2 samples before enzymatic degradation; (**C**) PCL and (**D**) PCL:MCC 98:2 samples after 4 h of enzymatic degradation; (**E**) PCL and (**F**) PCL:MCC 98:2 samples after 24 h of enzymatic degradation; (**G**) PCL and (**H**) PCL:MCC 98:2 samples after 48 h of enzymatic degradation. Scale bar: 300 µm. Magnification factor: ×300.

The observation of the SEM images (Figure 4) provides evidence about the effect of the MCC particles presence on the behavior of the enzymes while attacking the bulk material. As shown, the degradation occurs preferentially at specific sites, probably those with a higher concentration of filler, as its distribution is not homogeneous.

### 3.3. FTIR Spectra

Regarding the implications of the accelerated degradation of composite samples compared with pure PCL samples on the crystallinity of the bulk materials, it is worth mentioning the results of the FTIR characterization (Table 2). In this table, a higher ratio of the area of the CH_2_ peak to the area of the C–O–C peak is related to a lower degree of crystallinity [23]. As previously described, the introduction of the filler increases the initial crystallinity of the composite material (as shown by the values from Table 2: 1.73 for pure PCL and 1.66 for PCL:MCC 98:2), because the particles act as nucleating points during the crystallization process. This effect has been deeply analysed by Nerantzaki et al. [22], who studied the process quantitatively by the usage of the nucleation activity factor.

### 3.4. Numerical Method

Table 3 shows the results of the numerical method for pure PCL filaments. The computation time needed was 1531 s (approximately 25 min). Each row of Table 3 presents the simulated data of each iteration of the method. The first column shows the iteration number and the second column the volume of the boundary elements at the beginning of the iteration. In the third column is shown the reference removed volume according to the experimental data of mass loss (percentage values). Note that in this case, the density was considered a constant value, so that the mass loss percentage values and volume loss percentage values are identical. However, as the method works with volumes, it can easily deal with variable densities (the experimental mass and density data could be used to determine the loss volume, which is the reference parameter). The fourth column depicts the criterion applied (Monte Carlo or complete removal of all the boundary elements). As explained before, the complete removal of the boundary elements is applied only when the volume of the boundary elements is not enough to reach the reference data of removed volume. In those cases, the method removes the complete boundary directly. The fifth column shows the total removed volume (accumulated removed volume) at the end of the iteration. The sixth column depicts this same value, but in percentage with respect to the initial volume of the geometry. Therefore, the sixth and third columns should match if the method achieves a good fit with the experimental data. Precisely, the last column shows the deviation between these values, which is the difference between the total removed volume by the method and the reference removed value (experimental data) in percentage.

In the first iteration, the Monte Carlo criterion was applied, obtaining almost the desired degradation compared to the reference experimental value (only 0.01% deviation between the removed volume, 0.85%, and the reference value, 0.84%). Therefore, in the second iteration, the algorithm changes the reference removed volume to the following experimental data (6.16% of material loss). As the volume of the boundary elements is lower than the volume to be removed to reach this new reference value, the algorithm directly removes all the boundary elements. This way, in the third iteration, the algorithm applies again the Monte Carlo method with the adjusted amplification factor, achieving a good fit between the removed volume (6.17%) and the experimental reference value (6.16%). Therefore, the methodology converged in very few iterations to the reference values of mass loss. Note that the last reference value of mass loss for PCL (Table 1) was not included as input value in the numerical method since enzymatic saturation occurred.

As iteration 1 reached the first reference value of volume loss, the resulting mesh of this iteration corresponds to the degraded geometry at 4 h. Likewise, the resulting mesh of iteration 3 corresponds to the degraded geometry of the second reference value, at 24 h.

On the other hand, Table 4 shows the results corresponding to the composite filament (PCL:MCC 98:2). The computation time was 3549 s (approximately 59 min), which was higher than the time required for pure PCL (25 min). This difference in computation time is mainly due to the higher degradation rate and the longer degradation time of the composite filament (in PCL, the last degradation time was 24 h instead of 48 h, since the last experimental mass loss was not considered for the simulation). In this case, the first reference value of removed volume (1.12%) was reached in the first iteration, with the Monte Carlo method. However, the second reference was achieved after four additional iterations (three with the removal of all the corresponding boundary elements, and the last one applying the Monte Carlo method). This is due to the high difference between the first reference value of mass loss (1.12%) and the second one (13.68%). In fact, the complete removal of the boundary elements is useful for cases such as this, in which there is big differences between consecutive reference values of mass loss (the complete removal of the boundary elements allows a faster convergence to the experimental data). Finally, the third reference data of mass loss was achieved after two additional iterations with the Monte Carlo criterion (iterations 6 and 7). In iteration 6, the removed volume (16.13%) almost reached the corresponding reference value of mass loss (6.15%) (deviation of -0.02%). As the criterion established for the change of reference value (or end of the program if there are no more reference values) is not fulfilled (the deviation must be higher than −0.01% to change the reference value), the method applies another iteration with the Monte Carlo criterion (iteration 7). In that last iteration, it can be clearly observed the influence of the amplification factor in the probability function. In the previous iteration, the removed volume was the difference between 16.13% and 13.67% (2.46% of removed material in iteration 6). However, in the last iteration, the percentage of removed material was only 0.02% (16.15–16.13%). Therefore, it is demonstrated again that the amplification factor allows a very accurate fitting to the experimental results in a few number of iterations.

**Figure 5:**
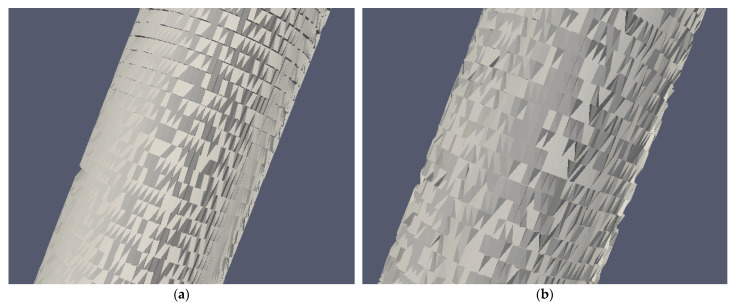
Detail of the degraded geometries: (**a**) PCL filament at 24 h; (**b**) PCL:MCC 98:2 filament at 48 h.

Figure 5 shows the degraded geometries of the PCL filament (after 24 h of degradation) and PCL:MCC 98:2 filament (after 48 h of degradation).

## 4. Discussion

The results shown in Table 1 demonstrate the ability of microcrystalline cellulose to increase the degradation rate of the matrix. In the literature, similar trends have been observed for other fillers, such as silk microparticles (enzymatic degradation) [21] or magnesium hydroxide (hydrolytic degradation) [37]. The increment in the mass loss can be explained by two simultaneous processes: the release of the microcrystalline cellulose particles while the degradation of the matrix takes place, and the improved degradation of the matrix itself as a consequence of the formation of voids inside the filament and an improved adhesion of the enzymes at the beginning of the process. The formation of the voids because of the release of the MCC particles allows water to diffuse inside the thermoplastic matrix and the cleavage of the ester bonds through hydrolysis takes place. As the diffusion of water is a key element on the explanation of the degradation kinetics [38], the increased surface exposed to water will definitely increase the degradation rate.

Regarding the mechanical behavior of the filaments over the degradation process, it was possible to confirm that the evolution of the elastic modulus vs. mass loss (Figure 3) is less abrupt in the case of the PCL:MCC samples: this group is able to provide a higher value of the elastic modulus than PCL samples at the same level of mass loss. This trend can be explained by the increased crystallinity of the composite material because of the nucleation effect provided by the particles of microcrystalline cellulose. This finding is a key issue of the present work as this characteristic could be useful in the development of parts for both the Tissue Engineering and the green packaging fields. In addition to this, the equations that correlate both parameters (elastic modulus and mass loss) are presented in this work in order to be used for simulation studies by the readers (also depicted in Figure 3).

Equation for pure PCL:

E = −0.8489 m^2^ − 1.3253 m + 306.38, (6)

Equation for the composite material:

E = −0.2552 m^2^ − 0.5031 m + 292.45, (7)

where E is the elastic modulus and m is the percentage of mass loss in percentage.

Regarding the degradation mechanism, the points with high concentration of the filler may promote enzyme adsorption and local degradation, as demonstrated in this work by the SEM images shown in Figure 4, where it is possible to observe the presence of large voids at points where degradation has been specifically activated. As stated above, the preferential degradation implies the release of particles of the filler at these points and, as a consequence, micropores are created. Afterwards, these micropores can improve water diffusion inside the material, promoting hydrolytic degradation from inside the micropores and an autocatalytic effect due to the liberation of carboxylic acids.

To explain this improved adsorption of the enzymes, it is possible to point out the importance of surface wettability on this type of processes [39]. In previous studies focused on the development of this composite material [40], the water contact angle was demonstrated to be decreased because of the presence of the filler. Surface changes may have a great impact on the ability of enzymes to be adsorbed. Enzyme adsorption is a key step on the mechanism of degradation in the presence of these catalysts. Therefore, a change on this step would dramatically affect the overall kinetics of the degradation process.

In addition to this, the increased hydrophilicity of PCL:MCC 98:2 composite material compared to pure PCL is also a characteristic that helps to improve the hydrolysis of the base material. As the first requirement for the hydrolysis of the polyester is the attack of the water molecules to the ester bonds, an increment of the hydrophilicity of the material will enhance the overall degradation process [41].

Regarding the FTIR spectra, it is possible to observe a decrease in the relative amount of amorphous phase in the degraded samples for both materials (Table 2), as enzymatic degradation is known to start in these domains of the polymer bulk [42] and it is then extended to crystalline domains [10]. However, this reduction is not continuous through the duration of the experiment and the ratio is slightly increased between 24 h and 48 h (from 1.57 ± 0.14 to 1.66 ± 0.05 for PCL samples and from 1.52 ± 0.01 to 1.61 ± 0.05 for composite samples). This change can be explained by the new crystallization of the segments of the macromolecular chains with a reduced molecular weight created during degradation. The short fragments are able to reorganize and create new crystalline domains, thus increasing the degree of crystallinity of the material. This trend is known as “degradation-induced crystallization” [6] and it was observed previously in PCL-based materials by Spearman et al. for their PCL-PGA nanocomposites [43].

As regards the numerical method presented, the approach is similar to the developed by Erkizia et al. [31], but with an adjustable parameter (amplification factor) that depends on the experimental results. This parameter allows a better fitting in fewer iterations. Moreover, the method applies the evaluation only in the surface elements, which is in line with the enzymatic degradation process (mainly a surface erosion since the enzyme molecules are sufficiently bulky to not be able to attack the polymer inside [15]). Therefore, the method degrades the geometry in accordance with the experimental results, thus allowing a good prediction of the degraded geometry.

On the other hand, it is worth mentioning that the density may not be constant during degradation. As the amorphous phase degrades faster than the crystalline phase, the crystallinity generally increases over degradation (unless degradation-induced crystallization takes place). As a result of this increased crystallinity, the density may also be higher over degradation. In this study, the density could not be measured with accuracy and was therefore considered a constant value. However, note that the proposed methodology works with volume properties, so that if the mass loss and density are known at every degradation time, the corresponding volume can be easily calculated (mass/density) and used as input array for the simulation.

Additionally, the results of the simulation could also be extrapolated to more complex geometries by adjusting the amplification factor. For example, for each iteration, the ratio of removed volume in that iteration with respect to the external volume (boundary elements in contact with the degradation media at the beginning of that iteration) could be used as a normalized amplification factor for other geometries with the same material and degradation conditions. This way, the simulation of the experimental geometry would serve as a training step to determine the normalized amplification factor of each iteration. Afterwards, for each iteration of the new geometry, the corresponding normalized amplification factor could be applied to adjust the degradation probability function. In other words, the initial simulation with the experimental geometry and guided by the experimental results would allow obtaining these normalized amplification factors, that could be applied for the degradation of other more complex geometries that may not be feasible to degrade due to the high costs of manufacturing and resources for the experimental tests. Moreover, the degraded geometries could be useful to carry out finite element analysis to predict the mechanical behavior after the degradation. This tool could be especially useful in the development of bioresorbable scaffolds for Tissue Engineering, as the degradation rate of the scaffold should match the regeneration rate of the native tissue. From the mechanical point of view, the objective is to maintain the overall mechanical performance of the set (scaffold and native tissue) relatively constant over time. In the initial stage, the scaffold will provide the most important mechanical properties of the set, but will gradually lose them as the native tissue grows. The native tissue, on the other hand, will compensate this loss by providing more mechanical properties to the set. Therefore, this tool could be useful to estimate the mechanical behavior of degraded scaffolds starting from empirical degradation data of more simple geometries.

## 5. Conclusions

The introduction of microcrystalline cellulose has been demonstrated to be a useful and simple strategy to modify the degradation rate of polycaprolactone matrices. This filler is released during the degradation process, creating new voids in the matrix that facilitate the adhesion of enzymes and water diffusion, thus accelerating the degradation rate. From the mechanical point of view, the mass loss is compensated in a certain way by the degradation-induced crystallization (reorganization of short fragments released during the process), leading to more crystalline zones and, as a consequence, lower reductions of the elastic modulus despite the high values of mass loss. Therefore, MCC filler in PCL matrices can be used to increase the degradation rate but keeping the elastic modulus more stable during the process.

The information presented in this work could be applied in different fields, including the design of composite polycaprolactone-based scaffolds and innovative eco-friendly materials, as the presence of the additive is able to increase the degradation rate of PCL-based composite materials, while keeping the mechanical properties more constant over time.

Regarding the numerical method presented for the estimation of the degraded geometry, the combination of the Monte Carlo algorithm with the adjusted probabilistic function (depending on the reference values of mass loss and the exposure of the elements to the degradation media) achieves a good agreement with the experimental data with a relatively low number of iterations. This method allows the prediction of the degraded geometry at the different degradation times. Moreover, the results of this simulation could be extrapolated to other geometries (with the same material and degradation conditions), thus allowing the estimation of the degraded geometry at different degradation times just with the initial training of the model, which can be performed in more simple and controlled geometries, such as filaments, and extrapolated to more complex geometries, such as scaffolds for Tissue Engineering. Further research will be carried out on this matter to investigate the capabilities of this method to extrapolate the results in other geometries. Additionally, in the specific case of Tissue Engineering applications, the proposed model for the enzymatic degradation, which is carried out in static conditions, could be coupled with Computational Fluid Dynamics to simulate more realistic conditions (body fluids).

## Figures and Tables

**Table 1:** Evolution of mass and elastic modulus with the enzymatic degradation time.

Material	Degradation Time (h)	Mass Loss (wt%)	Elastic Modulus (MPa)	pH
PCL	0	-	306 ± 46	7.59
PCL	4	0.84 ± 0.11	304 ± 29	7.00
PCL	24	6.16 ± 0.28	265 ± 37	6.28
PCL	48	5.88 ± 0.28	270 ± 25	5.59
PCL:MCC 98:2	0	-	295 ± 17	7.59
PCL:MCC 98:2	4	1.12 ± 0.15	288 ± 36	6.74
PCL:MCC 98:2	24	13.68 ± 1.31	239 ± 16	4.70
PCL:MCC 98:2	48	16.15 ± 5.28	216 ± 23	4.35

**Table 2:** Evolution of the FTIR peak areas (CH_2_ in the amorphous phase/C–O–C in the crystalline phase).

Material	Degradation Time (h)	Ratio of Areas (CH_2_ Peak at 2945 cm^−1^/C–O–C Peak at 1245 cm^−1^)
PCL	0	1.73 ± 0.05
PCL	24	1.57 ± 0.14
PCL	48	1.66 ± 0.11
PCL:MCC 98:2	0	1.66 ± 0.05
PCL:MCC 98:2	24	1.52 ± 0.01
PCL:MCC 98:2	48	1.61 ± 0.05

**Table 3:** Degradation results of the numerical method applied (PCL filament).

Iteration	Volume of Boundary Elements (mm^3^)	Reference Removed Volume (%)	Criterion Applied	Removed Volume (mm^3^)	Removed Volume (%)	Deviation (%)
1	4.62	0.84	Monte Carlo	1.21	0.85	0.01
2	5.45	6.16	Boundary elements	6.66	4.67	−1.49
3	5.10	6.16	Monte Carlo	8.80	6.17	0.01

**Table 4:** Degradation results of the numerical method (PCL:MCC 98:2 filament).

Iteration	Volume of Boundary Elements (mm^3^)	Reference Removed Volume (%)	Criterion Applied	Removed Volume (mm^3^)	Removed Volume (%)	Deviation (%)
1	4.62	1.12	Monte Carlo	1.60	1.12	0.00
2	5.59	13.68	Boundary elements	7.19	5.04	−8.64
3	5.11	13.68	Boundary elements	12.30	8.63	−5.05
4	4.38	13.68	Boundary elements	16.67	11.70	−1.98
5	4.81	13.68	Monte Carlo	19.49	13.67	−0.01
6	5.46	16.15	Monte Carlo	22.99	16.13	−0.02
7	4.84	16.15	Monte Carlo	23.02	16.15	0.00

## Data Availability

The data presented in this study are available within the article.
